# Obstructive sleep apnea in the hemodialysis population: are clinicians putting existing scientific evidence into practice?

**DOI:** 10.3389/fneph.2024.1394990

**Published:** 2024-06-10

**Authors:** David Andri Burkhalter, Antonio Cartellá, Domenico Cozzo, Adam Ogna, Valentina Forni Ogna

**Affiliations:** ^1^ Faculty of Biomedical Sciences, Università della Svizzera Italiana, Lugano, Switzerland; ^2^ Service of Pulmonology, Ente Ospedaliero Cantonale, Locarno, Switzerland; ^3^ Service of Nephrology, Ente Ospedaliero Cantonale, Locarno, Switzerland

**Keywords:** hemodialysis, sleep apnea, fluid overload, fluid management, nephrologist’s awareness

## Abstract

**Introduction:**

Hemodialysis (HD) populations have a high prevalence of Obstructive Sleep Apnea (OSA), which was specifically linked with fluid overload. HD fluid management targeting a low dry weight was shown to reduce OSA severity, opening to novel therapeutic options. We assessed nephrologists’ awareness of OSA diagnosis in HD patients and whether they integrate the current knowledge into their fluid management strategy.

**Material and methods:**

We performed a multicenter, cross-sectional study between July 2022 and July 2023, screening all HD patients of four HD units, and included those with confirmed OSA. We collected anthropometric parameters and fluid status from electronic dossiers. Predialysis fluid overload was measured by multifrequency bioelectrical impedance (BCM^®^). Nephrologists were asked to identify patients with known OSA, without consulting medical dossiers. The fluid management of patients identified as “OSA positive” was compared to that of patients misclassified as “OSA negative”.

**Results:**

Among 193 HD patients, 23.0% (n=45) had confirmed OSA. The mean age was 76.0 ± 7.5 years, 82.2% were men. Only 60% were correctly identified as “OSA positive” by nephrologists; 14.7% of patients on CPAP were identified. BMI was the only factor associated with correct OSA identification. The predialysis fluid overload tended to be greater in “OSA positive” patients than in the “OSA negative” patients (2.2 ± 1.4 kg vs 1.5 ± 1.3 kg; p=0.08), but there was no difference in postdialysis achievement of dry weight between the groups (residual overweight 0.2 ± 1.0 kg and 0.1 ± 0.7 kg; p= 0.672).

**Conclusions:**

Our study suggests that the application of scientific evidence to the management of OSA in dialysis patients is not systematic. However, nephrologists have attempted to strictly achieve dry weight in all patients, regardless of OSA status. Sensibilization of nephrologists on the clinical and diagnostic peculiarities of OSA in HD patients may improve OSA diagnosis and therapeutic care.

## Introduction

Obstructive sleep apnea (OSA) is a sleep-related respiratory disorder caused by overnight pharyngeal collapse, that results in recurrent episodes of breath interruption and desaturations during sleep. The clinical manifestations are snoring, frequent awakenings with disrupted sleep and excessive daytime sleepiness (1). The amplified oxidative stress and overdrive of the sympathetic nervous system determined by repeated oxygen saturation drops explain the increased cardiovascular risk observed in OSA patients (2, 3).

Over the last 10 years, the amount of scientific evidence concerning sleep apnea in end-stage kidney disease (ESKD) patients has grown steadily. In 2015, a cross-sectional multicenter study showed that OSA prevalence was significantly greater in a chronic intermittent hemodialysis (iHD) population in the French-speaking area of Switzerland than in the general population in the same geographical area., suggesting the existence of pathophysiological mechanisms specific to this population (4, 5).

The high prevalence of OSA in chronic iHD patients was meanwhile linked to fluid overload, which promotes overnight upper airway collapse through fluid accumulation in the parapharyngeal tissue (6–8). An intensified fluid removal strategy during iHD treatment was found to attenuate the severity of OSA in iHD patients, leading to new therapeutic options in this population (9, 10).

Considering the high prevalence, cardiovascular consequences and therapeutic possibilities (continuous positive airway pressure, fluid overload optimization), OSA screening should be considered part of the usual workup for iHD patients.

Nevertheless, OSA was largely underdiagnosed and undertreated in the reported iHD cohort. Furthermore, classical OSA screening tools, in the form of questionnaires or scores, showed poor sensitivity and specificity for OSA diagnosis in this population (4, 11).

The aims of this study were 1) to assess the prevalence of confirmed OSA in an ESKD population undergoing iHD in the Italian-speaking area of Switzerland, 2) to assess the awareness of treating nephrologists about the OSA diagnosis in their patients, and 3) to evaluate whether nephrologists integrate the current knowledge into their fluid management strategy for iHD patients with OSA.

## Materials and methods

### Study protocol

We conducted a multicenter, cross-sectional population study. Between July 2022 and July 2023, we screened all patients attending the HD units of four regional hospitals of Ente Ospedaliero Cantonale (EOC), the public hospital network in Italian-speaking Switzerland. Patients who fulfilled the inclusion criteria (age ≥18 years, chronic iHD for more than three months, confirmed OSA according to medical records) were included in this study if they consented to the use of their data for research purposes through institutional informed consent. The study complied with the Declaration of Helsinki and was approved by the Institutional Ethics Committee (Comitato Etico del Canton Ticino, Bellinzona, Switzerland).

### Data collection

Data pertaining to the patients’ medical history, anthropometric parameters, fluid status and HD protocol characteristics were collected from local electronic patient dossiers. Specifically, patient records were manually scanned for any mention of OSA or sleep related-breathing disorders, any previous sleep breathing recording using polygraphy (PG) or polysomnography (PSG), and any mention of CPAP treatment.

Patients with suspected but not investigated OSA and patients with negative sleep recording results (apnea hypopnea index, AHI < 5/h) were considered to not have OSA.

Predialysis fluid overload was obtained from multifrequency bioelectrical impedance measurements. In the four HD units, bioimpedance measurements are routinely conducted (every 3 to 4 months) before a given dialysis session to evaluate fluid overload using Body Composition Monitor^®^ (BCM^®^, Fresenius Medical Care, Bad Homburg, Germany). This technique has been validated in ESKD patients on iHD (12, 13).

For each included patient, we collected the following data, which were tracked on the day of the BCM^®^ recording: height, pre- and postdialysis weight, nephrologist-defined target weight (i.e., ideal weight defined by the nephrologist), predialysis fluid overload (assessed by BCM^®^) in absolute values and percentages of extracellular water and finally BCM^®^-defined normal hydrated weight. Body mass index (BMI) was calculated as postdialysis weight divided by height in square meters.

### Nephrologists’ interview

Nephrologists in charge of the HD units were asked to identify patients in their HD units with known OSA diagnoses, without consulting medical dossiers. They were also questioned about patients using treatment with CPAP and were interviewed about their clinical practice, asking them if they “were used to adapt HD treatment or apply individualized therapeutic measures in patients with known OSA”. In this second part of the interview, we included two additional nephrologists.

### Statistical analysis

To analyze the impact of OSA on the treatment choices of nephrologists, the included patients were divided into two groups according to their awareness of OSA among nephrologists: patients correctly identified as “OSA positive” and those misclassified as “OSA negative” despite confirmed OSA.

We performed descriptive statistics to present the collected data as the mean ± standard deviation for continuous variables and as absolute numbers and percentages of available observations for categorical variables. We used t-tests, chi-square tests and Fisher exact-tests to compare the characteristics of the patients between the groups. A logistic regression model was fitted to assess the association between various demographic and clinical variables and the awareness of OSA among nephrologists. p < 0.05 indicated statistical significance.The statistical analysis was performed using R version 4.3.1 (R Core Team 2023, R Foundation for Statistical Computing, Vienna, Austria; *https://www.R-project.org/* ).

## Results

### Study population

Out of 193 patients treated with iHD in the four HD units, 45 patients with a confirmed diagnosis of OSA were included in this study. The demographic and medical data of the studied population are detailed in **Table 1**.

**Table 1:** Characteristics of the study population.

	Mean ± SD or N (%)
**Age (y)**	76.0 ± 7.5
**Male gender**	37 (82.2%)
**Caucasian ethnicity**	44 (97.8%)
**BMI (kg/m^2^)**	29.1 ± 4.5
**Kidney disease:** - **Diabetic** ** - Hypertensive** ** - Glomerulonephritis** ** - Others/Unknown**	16 (35.5%) 10 (22.2%) 6 (13.3%) 13 (28.9%)
**Previous kidney transplantation**	2 (4.4%)
**Diuresis > 300 ml/24h**	25 (55.6%)
**Residual diuresis (L)**	1.03 ± 0.44
**Vascular access** ** - Fistula** ** - Catheter**	28 (62.2%) 17 (37.8%)
**Hemodiafiltration**	23 (51.1%)
**HD treatment time (h/week)**	11.3 ± 1.1
**Dialysis dose (eKt/V)**	1.33 ± 0.25
**Hemoglobin (g/l)**	101.5 ± 15.1
**Mid-week HD session**	31 (68.9%)
**Pre-dialysis systolic BP (mmHg)**	146 ± 22
**Pre-dialysis diasotlic BP (mmHg)**	70 ± 13
**Pre-dialysis fluid overload according to BCM*^®^* (L)**	2.4 ± 1.7
**Pre-dialysis fluid overload according to BCM*^®^* (% of ECW)**	16.2 ± 6.7
OSA severity*: ** - Mild (AHI ≥ 5/h < 15/h)** ** - Moderate (AHI ≥15/h <30/h)** ** - Severe (AHI ≥ 30/)**	8 (19.0%) 6 (14.3%) 28 (66.7%)
**CPAP treatment**	34 (75.5%)
**Comorbidities:** ** - Diabetes** ** - Chronic Heart Failure** ** - Coronary Heart Disease**	33 (73.3%) 17 (37.8%) 18 (40.0%)

*missing data from 3 participants (N=42).

BCM^®^, Body Composition Monitor; BMI, body mass index; OSA, obstructive sleep apnea; AHI, apnea-hypopnea index; BP, blood pressure; CPAP, continuous positive air pressure; ECW, extracellular water; HD, hemodialysis; eKt/V, hemodialysis efficacy assessed using urea kinetic modelling.

**Figure 1:**
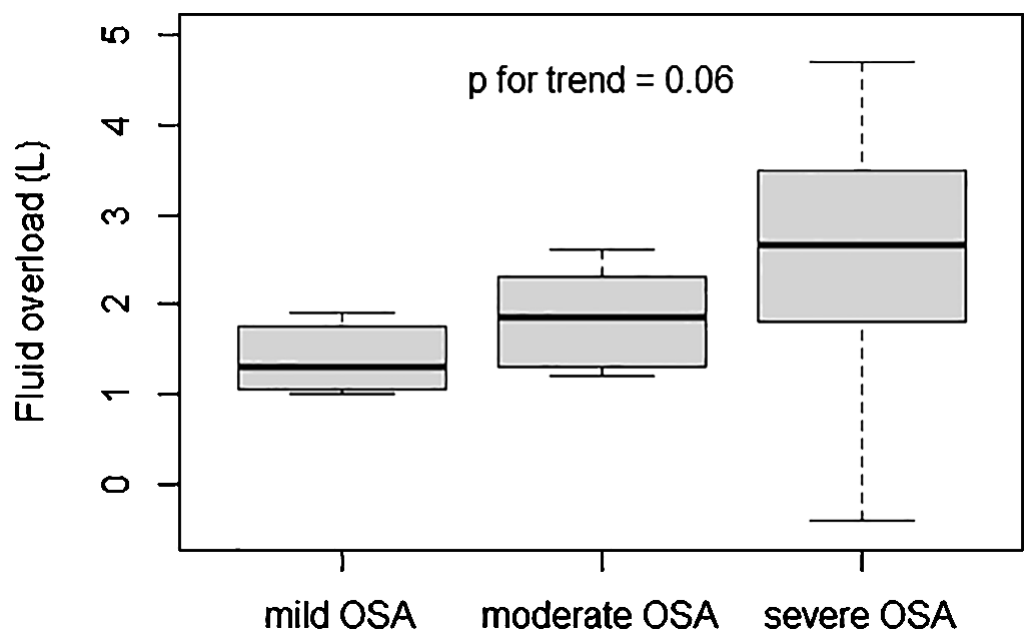
Association between OSA severity and fluid overload. Fluid overload: difference between pre-HD weight and BCM^®^-defined normal hydrated weight; OSA, obstructive sleep apnea.

The prevalence of diagnosed OSA in the study population was 23.0%. Thirty-four patients were treated with CPAP, representing 75.5% of the OSA patients. There was a trend toward a positive association between pre-HD fluid overload and OSA’s severity (p = 0.063) (**Figure 1**).

### OSA *awareness* among *nephrologists*

**Figure 2:**
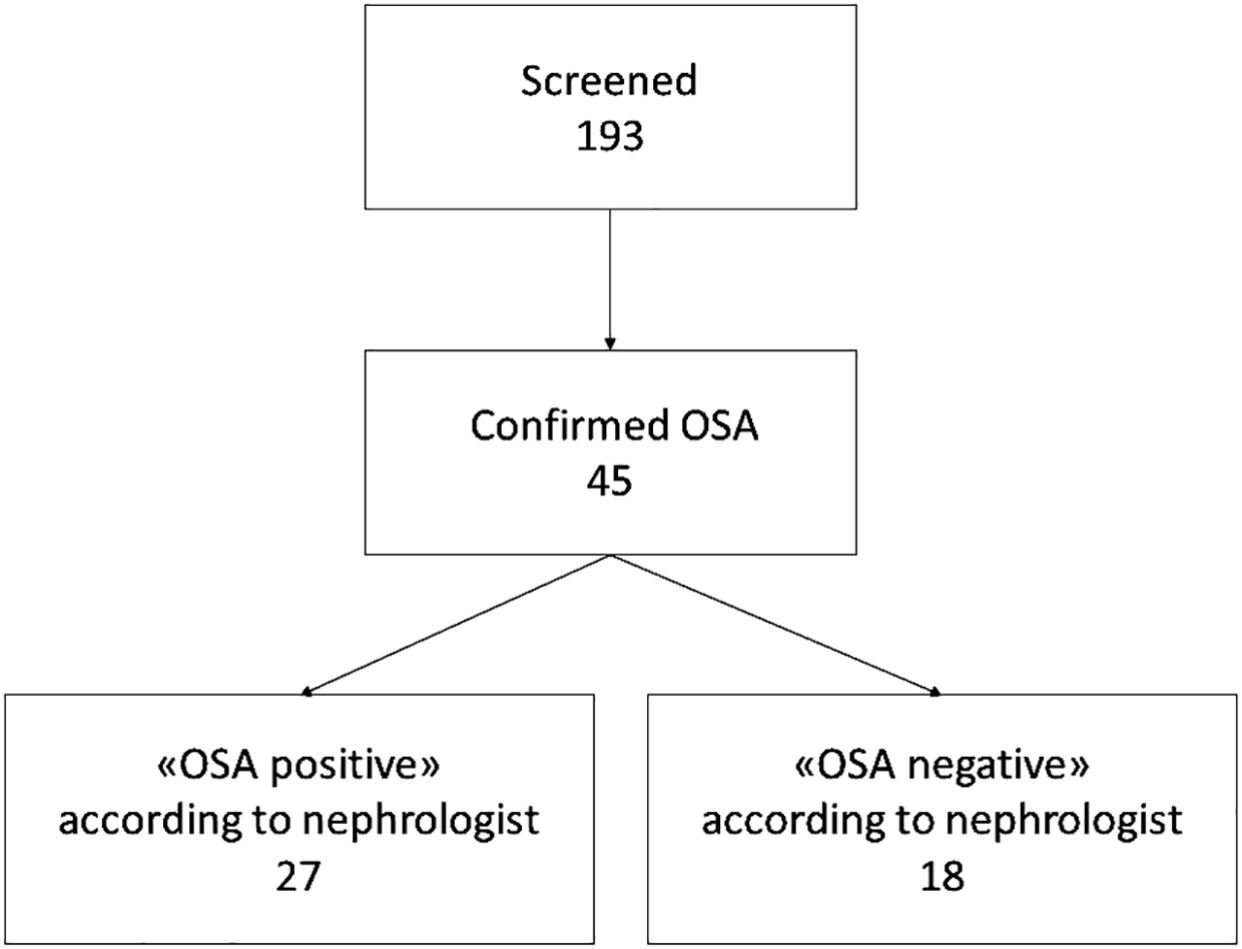
Study flow chart. OSA, obstructive sleep apnea.

Of the 45 patients diagnosed with OSA, only 27 (60.0%) were correctly identified by the treating nephrologist (**Figure 2**). We observed no difference in OSA awareness between the nephrologists in charge of the four participating HD units (p = 0.964).

Concerning CPAP treatment, only five patients (14.7%) were correctly identified by the interviewed nephrologists.

Studying the clinical characteristics that were associated with the awareness of OSA among nephrologists, only high BMI (p=0.003) was shown to predict the accuracy of OSA diagnosis, whereas age, sex, OSA severity and/or CPAP therapy were not.

According to the second part of the interview, only half of the nephrologists (4/8) were aware of the pathophysiological link between fluid overload and OSA, and two of them reported to integrate this knowledge into their fluid management strategy, even if not systematically. **Table 2** resumes the results of the nephrologist’s interview.

**Table 2:** Nephrologist’s interviews.

Nephrologist	Do you adapt HD treatment or apply individualized therapeutic measures in patients with known OSA?	Which specific measure would you adopt?
1 – no HD nephrologist	No	None
2 – no HD nephrologist	Yes	Avoid fluid overload
3 – HD nephrologist	No	Avoid acid-base disbalance
4 – HD nephrologist	No	Avoid fluid overload
5 – HD nephrologist	No	None
6 – HD nephrologist	Yes	Avoid fluid overload
7 – HD nephrologist	No	Avoid acid-base disbalance
8 – HD nephrologist	No	Avoid fluid overload

HD, hemodialysis; OSA, obstructive sleep apnea.

### Fluid management of OSA patients

We found no difference in the weight target defined by the treating nephrologist between the patients correctly identified as “OSA positive” and those misclassified as “OSA negative”, both being close to the normal hydrated weight calculated with BCM^®^ (mean difference 0.7 ± 2.3 and 0.3 ± 1.4 kg respectively; p= 0.564).

The predialysis fluid overload (according to the nephrologist’s targeted dry weight) of the whole population was 1.9 ± 1.4 l (16.2 ± 6.7%), tending to be greater in the “OSA positive” group (2.2 ± 1.4 l and 16.8 ± 8.0%) than in the “OSA negative” group (1.5 ± 1.3 l and 15.4 ± 4.3%; p=0.08 and p = 0.08 respectively).

**Figure 3:**
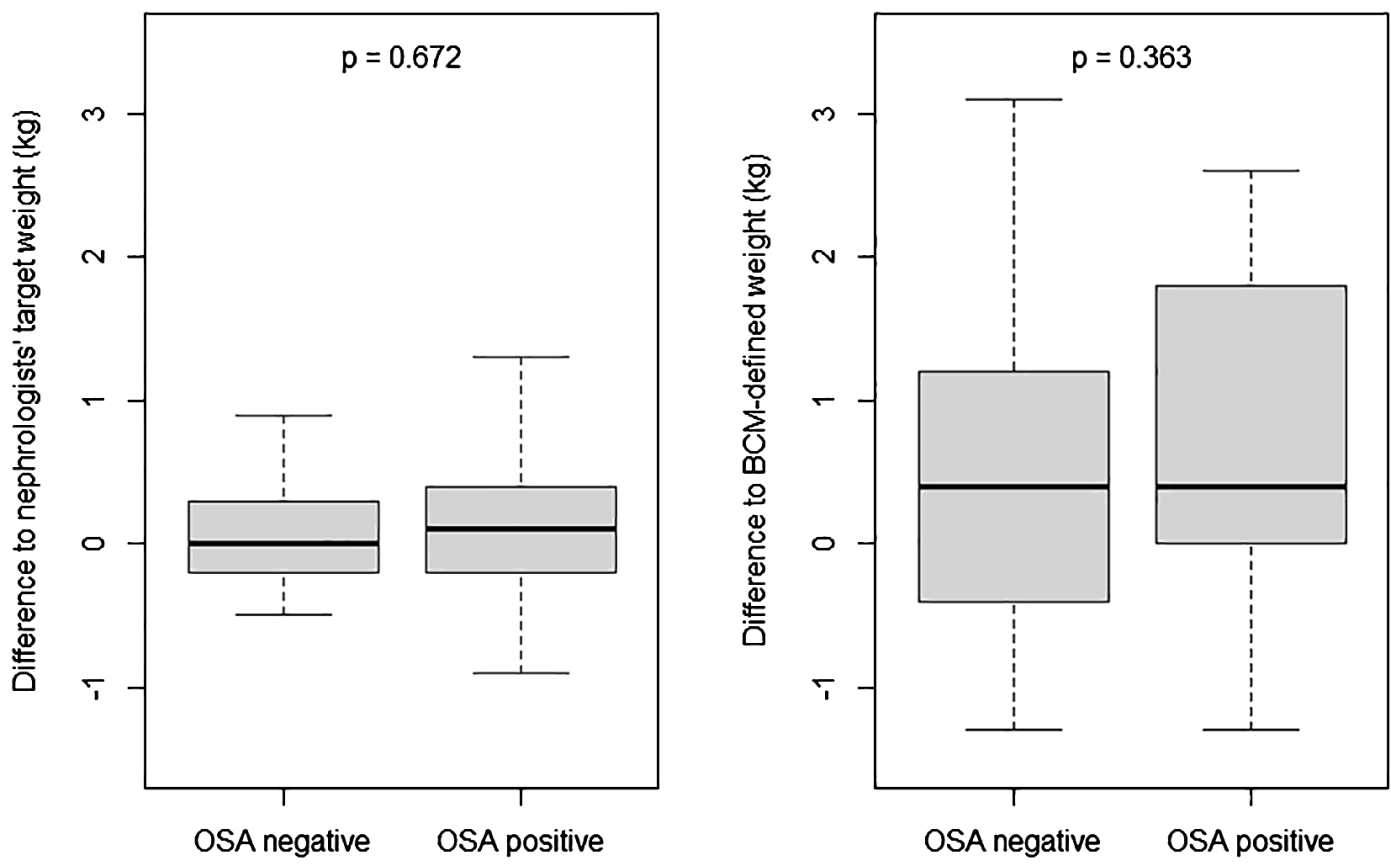
Difference between measured post-HD weight and target weight. HD, hemodialysis; OSA, obstructive sleep apnea; “OSA positive”: patients with already known OSA correctly identified by the treating nephrologists; “OSA negative”: patients with OSA misclassified as not having OSA by the treating nephrologists. The 0 value on the Y-axis represents the target dry weight defined by the nephrologist (left graph) or the BCM^®^-defined normal hydrated weight (right graph).

Despite this difference in the predialysis weight, the achievement of nephrologist’s target weight after HD was equal between the two groups (residual overweight 0.2 ± 1.0 vs 0.1 ± 0.7 kg; p= 0.672), without differences between HD units (p = 0.896). Also the differences between the post-HD weight and the BCM-defined normal hydrated weight were similar (0.9 ± 2.0 vs. 0.4 ± 1.7; p=0.363) (**Table 3** and **Figure 3**).

**Table 3:** Weight values of the two study groups.

	“OSA positive” (N=27)	“OSA negative” (N=18)
**Predialysis weight (kg)**	94.24 ± 14.76	74.24 ± 11.57
**Postdialysis weight (kg)**	92.25 ± 14.41	72.88 ± 11.49
**Nephrologist’s defined target weight (kg)**	92.05 ± 14.41	72.78 ± 11.53
**BCM^®^-defined normal hydrated weight (kg)**	91.39 ± 14.76	72.44 ± 11.75

OSA, obstructive sleep apnea; BCM, Body Composition Monitor.

## Discussion

The results of our cross-sectional population study suggest a scarce awareness of nephrologists about the potential role of OSA in iHD patients, regarding both the prevalence of the disease, and its potential consequences on the treatment strategies.

The prevalence of diagnosed OSA in our study population was less than half of the prevalence found in the French Switzerland iHD cohort (23.0% vs 56%, p< 0.001) (4) and in other similar populations investigated with systematic OSA screening. A recent meta-analysis described a prevalence of 55% (95% CI 47%–63%), pooling 42 studies performed using sleep monitoring devices (14).

The discrepancy between our prevalence results and the abovementioned values suggests that OSA is underdiagnosed in our iHD centers. This can be explained by the absence of a systematic screening for OSA, as the decision to screen was left to the clinician. Furthermore, our observation reveals the difficulty of nephrologists in discriminating individuals with OSA among patients in their care, with only two out of three patients with an OSA diagnosis reported in their medical history being identified and even fever patients using CPAP. However, dialysis patients are mostly polymorbid individuals with complex medical histories, which can hamper physicians’ knowledge of the entire diagnostic list (15).

Only a few studies have reported the magnitude of OSA underdiagnosis in the iHD population, comparing the diagnosed OSA cases with the real OSA prevalence, determined by systematic sleep recording of the whole population. Jurado-Gamez et al. found a prevalence of 44% in an iHD population of 32 patients by polysomnographic (PSG) screening; only 38% of these patients had been previously identified (16). In the already cited study on the French Switzerland cohort that included 104 iHD patients, only a small proportion of all OSA patients (19%) had been previously diagnosed, and even fever (10%) had been treated (4).

Some specificities of OSA in end-stage kidney disease (ESKD) could be responsible for the observed OSA underdiagnosis in this population. Clinical suspicion of OSA based on symptoms or clinical characteristics seems particularly disappointing in the ESKD population. As a consequence, the classical screening scores for OSA (Berlin Questionnaire, STOP-BANG and Adjusted Neck Circumference), which were developed and validated in the general population, showed poor performance in ESKD patients (4, 11). According to the abovementioned meta-analysis of 2023, the pooled OSA prevalence in the 28 studies based on sleep questionnaires was considerably lower than the prevalence obtained with sleep recordings (39% vs 55%) (14).

The OSA screening tools repose on the clinical characteristics associated with OSA in the general population, such as sleep-related symptoms (excessive daytime sleepiness, snoring, witnessed apneas, nocturnal choking), obesity and hypertension. However, ESKD patients are less likely to present with the stereotypical features of OSA. Specifically, daytime sleepiness does not seem to predict the presence of OSA; fatigue is a common complaint of ESKD, regardless of underlying sleep-disordered breathing; and hypertension is very common and often inadequately controlled in HD patients (17–20).

In our cohort, only high BMI was associated with awareness of OSA diagnosis by the treating nephrologists. According to the literature, however, a higher BMI was associated with more severe OSA in non-dialysis ESKD participants but not in iHD participants (4, 19). Previous studies have shown that OSA is associated with age, neck circumference, total body extracellular fluid volume and time on renal replacement therapy in HD patients (4, 10). Although classical factors do not seem to be useful in identifying patients at risk, no clear discriminating factors emerge from the literature.

The combination of these factors may explain the poor ability of nephrologists in identifying patients at risk of OSA (worthy to be referred for screening with sleep monitoring devices) and also patients with already diagnosed OSA.

Current scientific knowledge clearly supports the role of fluid overload in the pathogenesis of OSA in ESKD patients with chronic fluid overload (9). The underlying pathophysiological mechanism was identified in the overnight rostral fluid shift (i.e., fluid displacement occurring overnight from the legs to the neck soft tissues), leading to a reduction in the cross-sectional area and increased collapsibility of the upper airways, predisposing patients to OSA (6–8, 21).

There is growing evidence that targeting fluid overload by ultrafiltration/hemodialysis is an effective option for counteracting overnight rostral fluid shift and may lead to a reduction in OSA severity in patients with ESKD receiving renal replacement therapy.

In the early 2000s, Hanly et al. demonstrated that an intensification of fluid removal through the conversion from thrice-weekly iHD to daily nocturnal HD six-times per week led to a 68% reduction in OSA severity and to an improvement in fluid overload (22). Similar results were obtained in peritoneal dialysis patients by converting the technique from 24-h continuous ambulatory to nocturnal peritoneal dialysis, the latter of which allows intensified fluid removal (23).

Almost 10 years ago, Lyons et al. showed that removing an average of 2.2 L of fluid by isolated ultrafiltration in a single HD session reduced OSA severity by 36% (AHI from 43.8 ± 20.3 to 28.0 ± 17.7; p < 0.001) (10). In an interventional study performed in the same period, a significant correlation between the change in fluid overload volume after HD and the change in OSA severity (ΔAHI: 10.1 +/- 10.8/h) was demonstrated, regardless of metabolic parameters or HD efficacy (24). Moreover, the subgroup with lower fluid overload after the HD session had significantly lower OSA severity.

Given the contribution of fluid overload to the pathogenesis of OSA in ESKD patients, establishing and maintaining dry weight is of particular importance when managing ESKD patients with OSA.

In the present study, we found no difference in the fluid management strategy used for patients considered by nephrologists as “OSA negative” versus patients correctly identified as “OSA positive”. Specifically, 1) the target weight defined by the treating nephrologist and the BCM^®^-defined normal hydrated weight were similar in both groups, and 2) the weight achieved at the end of the HD session was very close to the dry weight set by the nephrologist.

These results reflect the efforts of nephrologists to optimize the fluid volume status of all patients, regardless of the diagnosis of OSA.

In support of proper management of the fluid balance, the fluid volume status values measured by bioimpedance were similar to those observed in an interventional study, including a similar population of OSA patients on maintenance iHD (24).

Moreover, clinical evaluation of fluid status in dialysis patients lacks sensitivity and specificity. Bioimpedance analysis is widely accepted as an objective method for determining body composition and the degree of fluid overload in patients with chronic iHD and has become part of the clinical practice of many dialysis centers worldwide (13). A BCM^®^-guided fluid management strategy was associated with better management of fluid status than was routine clinical judgment, leading to improvements in cardiovascular endpoints, such as regression of left ventricular mass index, a decrease in blood pressure, and improvements in arterial stiffness (25, 26).

Analogous to what has been described for these volume-dependent outcomes, it is conceivable that a BCM^®^-guided fluid strategy could improve the severity of OSA in the long-term through fluid status optimization. To date, however, no interventional study has been performed to substantiate this hypothesis.

There are several limitations of our study that need to be considered. First, we did not calculate the sample size, but screened all patients attending the four HD units.

Second, we did not perform a systematic instrumental screening of OSA; instead, we included patients with already established diagnoses according to medical records. The low prevalence we observed suggests that we have incurred selection bias. Since we did not screen the whole iHD population, we chose not to compare the study population with the rest of the iHD population since we did not know their OSA status. Including all patients across the centers would have broadened the insights, for example, allowing us to compare the fluid volume status of the two groups. Finally, we limited the analysis to the fluid volume status values of a single dialysis session.

## Conclusions

Nephrologists’ sensitivity to sleep apnea screening in HD patients seems to be low, despite its high prevalence in this specific population and the available scientific evidence supporting that targeting fluid overload by ultrafiltration/hemodialysis is an effective option for improving OSA severity.

Sensibilization of nephrologists on the clinical and diagnostic peculiarities of OSA in patients with ESKD may improve OSA diagnosis and therapeutic care.

## Data availability statement

Derived data supporting the findings of this study are available from the corresponding author VF on request. Requests to access these datasets should be directed to valentina.forniogna@eoc.ch.

## Ethics statement

The studies involving humans were approved by Comitato Etico del Canton Ticino, Bellinzona, Switzerland. The studies were conducted in accordance with the local legislation and institutional requirements. The participants provided their written informed consent to participate in this study.

## Author contributions

DB: Conceptualization, Data curation, Investigation, Methodology, Validation, Writing – original draft, Writing – review & editing. AC: Formal analysis, Investigation, Validation, Writing – original draft, Writing – review & editing. DC: Investigation, Validation, Writing – original draft, Writing – review & editing. AO: Formal analysis, Methodology, Validation, Visualization, Writing – original draft, Writing – review & editing. VF: Conceptualization, Data curation, Formal analysis, Investigation, Methodology, Project administration, Resources, Software, Supervision, Validation, Visualization, Writing – original draft, Writing – review & editing.
